# Blood Parameters in Treatment with Arsenic Trioxide in Acute Promyelocytic Leukemia: *A *Systematic Review

**Published:** 2020-04-01

**Authors:** Mehdi Mohammadi Kanesbi, Lida Jarahi, Mohammad Reza Keramati

**Affiliations:** 1 Cancer Molecular Pathology Research Center, Mashhad University of Medical Sciences, Mashhad, Iran; 2 Department of Community Medicine, Faculty of Medicine, Mashhad University of Medical Sciences, Mashhad, Iran

**Keywords:** Acute promyelocytic leukemia, Arsenic trioxide, All-trans retinoic acid, Chemotherapy, Disseminated intravascular coagulation

## Abstract

Acute promyelocytic leukemia (APL) is a subtype of acute myeloid leukemia (AML). APL is famed with some special blood coagulation disorders such as disseminated intravascular coagulation (DIC). The therapeutic methods of APL contain All Trans Retinoic Acid (ATRA), arsenic trioxide (ATO) or/and chemotherapy. Many studies have been done on APL blood disorders and its treatment. These studies have shown different results. In this systematic article, we tried to review the effect of ATO therapy with or without ATRA and chemotherapy on DIC parameters (D-dimer, Prothrombin Time, Activated Partial Thrombin Time, Platelet count) in APL patients. The result of included studies demonstrated that although ATO can reduce the number of malignant cells in the bone marrow and peripheral blood, it does not have enough potential to attenuate the danger of high score DIC that is usual in APL patients and should be better to be used with other therapeutic methods.

## Introduction

 Acute Myelocytic leukemia (AML) is the most common acute leukemia in adults^   1 ^. The AML is classified into two categories. The first one is FAB that known as FRENCH-AMERICAN-BRITISH which is dividing AML into some characterized groups (AML-M0-AML-M7). The newest classification of AML belongs to WHO that classifies AML by mutations occurring in each class.

Acute promyelocytic leukemia (APL) happens in 10-15% of AML incidence ^   2 ^. 98% of APL patients involve with the reciprocal and balanced translocation (15;17) (q22;q21)(PML-RARA) that include PML gene on chromosome 15 and RARA on chromosome 17. Acute promyelocytic leukemia is resulted by the halted myeloid precursors at the promyelocytic stage^   3 ^. Morphology of peripheral blood smears (PBS) in APL blood samples indicates more than 20% blasts with promyelocytic dominance, and usually there are some inclusions in the cytoplasm of promyelocytes named Auer rods in bone marrow aspiration or peripheral blood sample^   4 ^. In addition to these, APL has some other complications such as a high incidence of early hemorrhagic deaths^   5 ^. 90% of APL patients have hemorrhage disorders^   6 ^. A high rate of early death (about 30-60%) is due to the severe hemorrhagic disorders^   7 ^. For that, the best way to save patients from more aggravate conditions or death is treating them as a medical emergency^   5 ^. The hemorrhagic disorder forms are disseminated intravascular coagulation (DIC), thrombosis and bleeding ^   8 ^^,^^   9 ^^,^^   10 ^. Tissue factor releasing from the APL cells is considered to be the most important reason for the coagulopathy. The other significant cause of DIC occurrence in APL is the secretion of proteolytic enzymes from primary cytoplasmic granules from APL cells. These enzymes release in the blood, so they can cause both microvascular damage and bleeding induction^   11 ^. In these events, evaluations of some factors are necessary to be done (i.e. D-dimer level, Prothrombin time, Thrombin time, Fibrinogen concentration)^   8 ^. Different types of therapies could be done to restrict the progression of APL disease. The present study tries to discuss laboratory outcomes of DIC parameters [D-dimer, Prothrombin Time (PT), Activated Partial Thrombin Time (APTT) and Platelets (PLT)] and White blood cell (WBC) before and after therapy in various therapeutic methods. Therapy should include arsenic trioxide (ATO) with or without other treatment. 

**Diagnosis of APL**

If the patient is suspected for AML, the first stage is seeking the Peripheral Blood Smear (PBS) or bone marrow aspiration in order to calculate the blasts or promyelocytes percent. The AML is recognized when the count of blasts or precursors is 30% in FAB classification or 20% in the WHO classification of whole blood cells or bone marrow aspiration. For more confidence in the results, CD markers typing by flow cytometry, cytogenetic and karyotype method are appropriate to be done. CD markers which express on the APL cells include: CD33+, CD13+, CD34+, CD10+, ANTI-MPO+ and CD7-, CD79a-, CD117-, CD19-^4^. In molecular genetics, the presence of t(15;17)( PML-RARA) hybrid gene is observed^   12 ^.

## MATERIALS AND METHODS

**Figure 1:**
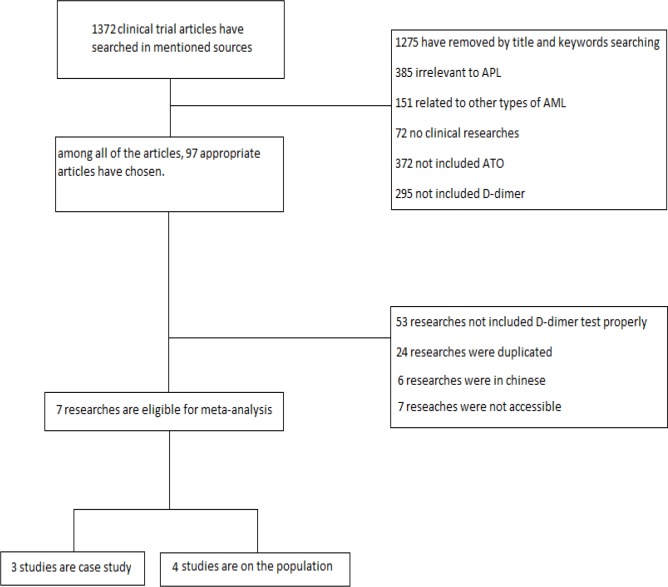
Whole eligible related studies involved in the article

**PICO**

P (Patients): This study included acute promyelocytic leukemia

I (Intervention): it has been tried to select studies that patients consumed the arsenic trioxide with or without all-trans retinoic acid or chemotherapy.

C (Comparison): Patients’ status between before and after therapy and differences between DIC parameters test results.

O (Outcome): Determination of ATO effect on the DIC parameters (D- dimer, PT, APTT, Platelet) and WBC count.

**Data extraction and analysis**

This study was done in 2019 at Mashhad University of Medical Science, Mashhad, Iran. The data are extracted from the mentioned sources (PUBMED, SCOPUS, EMBASE, and CLINICALKEY). In the search strategy process, our focus was on the clinical articles on APL patients which contained coagulation tests and blood cell count measurement. The sex, age, and the number of recurrence of disease in the patients were not considered. Data were analyzed by *REVMAN* ver.5 software.

**Inclusion criteria**

[A]: patients with APL disorder [B]: DIC parameters measurement in APL patients before the initiation of the therapy [C]: the presence of the arsenic trioxide in the process of treatment [D]: DIC measurement after the treatment course [E]: clinical study articles on APL cases.

**Exclusion criteria**

[A]: patients with other types of acute myeloid leukemia (non-APL) [B]: studies without DIC parameters measurement [C]: studies without arsenic trioxide in therapy method [D]: review, systematic or other non-clinical studies.

**Table 1:** Diagnosis criteria of overt DIC by the ISTH and the KSTH    (11)

**Variable**	**Overt DIC by ISTH**	**Overt DIC by KSTH**
Platelet	50,000-100,000/µl: 1 point ˂50,000/µl: 2 points	˂100,000/µl: 1 point
PT/APTT	Prolongation of PT: 3-6 sec: 1 point ˃6 sec: 2 points	Prolongation of PT: ˃3 sec: 1 point Prolongation of aPTT˃5 sec: 1 point ˃6 sec: 2 points
Fibrinogen	100 mg/dl: 1 point˂	150 mg/dl: 1 point˂
D-dimer	0.5-1 µg/ml: 1 point 1-2 µg/ml: 2 points ≥2 µg/ml: 3 points	
Total	Overt DIC ≥ 5 points	Overt DIC ≥ 3 points

DIC: Disseminated Intravascular Coagulation, ISTH: International Society and Thrombosis and hemostasis, KSTH: Korean Society Thrombosis and Hemostasis, PT: Prothrombin time, APTT: Activated Partial Thromboplastin Time

**Therapy**

Early hemorrhagic deaths in APL mainly occur due to the severe thrombo-hemorrhagic coagulopathy^   9 ^. Patients with severe thrombocytopenia (Platelet ≤20000/µl) should receive one/more platelet transfusions. Because of the coagulopathy disorders, the amount of fibrinogen decreases and on the other side, Prothrombin Time (PT) and Activated partial thromboplastin time (PTT) increase. Cryoprecipitate and FFP can increase the rate of fibrinogen and other coagulation factors^   4 ^.

**All-trans retinoic acid (ATRA)**

This drug is a famous therapeutic method in APL patients. The mechanism of ATRA is the induction of progress in cell differentiation and converts promyelocytes to the Band/Neutrophils. Besides the induction of cell differentiation, ATRA decreases the expression of the TF factor and cancer procoagulant. Furthermore, ATRA has some more protective effects such as 

neutralizing cytokines effects, augmentation in thrombomodulin, a decrease in TF upregulation and endothelial cells maintenance^   11 ^. Although ATRA controls the bleeding and this is a major advantage of this drug, thrombosis is a significant complication of ATRA ^4^^,^^13^ . Hung Chang et al. study claimed that delaying in APL treatment with ATRA causes severe hemorrhagic events in involved patients^4^. Lou Y et al. study demonstrated that delaying in the treatment with ATRA significantly increases the rate of mortality^   13 ^. It is important to mention that the most common sites of hemorrhage in APL patients are lungs and brain^4^^,^^14^. The combination of ATRA and other anti-cancer therapies caused 90% of complete remission (CR) in APL patients. Although ATRA is the first step of therapy for the APL, this drug has some side effects. The side effects of ATRA are such as hypertension, cardiac failure, pericardial effusion or pleural effusion, headache and arthralgia^   6 ^. ATRA as a single agent therapy has a 50-80% CR rate in APL patients^27^. 

**Arsenic trioxide (ATO/AS2O3)**

The major role of ATO is the induction of apoptosis and differentiation in the leukemic cells^15^^,^^16^^.^ This drug is an effective path in ATRA refractory patients^   17 ^. Although ATO cannot return the parameters to normal but disappears the hemorrhagic symptoms after 1-2 weeks after treatment ^   17 ^. The study on CD11b+ granulocytes and NBT cells in Shen et al. study revealed that the effect of ATO is weaker than ATRA on cell differentiation field (92.72%±19.6% and 56.9%±12.9% compared with 15.0%±4.7 and 10.7%±3.9% in the control for ATRA and 29.4%±9.1% and 23.4%±8.7% for ATO)^   18 ^.

The Mechanism of apoptosis induced by arsenic trioxide includes various types of mitochondrial damages which cause caspase activation and releasing cytochrome C (CYT-C) into the cytosol, so then it binds and activates APAF-1. Finally, CYT-C+APAF-1 complex activates procaspase-9. Caspase 9 cleaves procaspase 3, 6 and 7. This process prepares the cell for the internal apoptosis pathway^   20 ^. 

## Results

 All patients in selected studies were involved with t(15;17)(PML-RARA) because the incidence of coagulopathies is higher in this subtype of leukemia. The studies in Tables 2-7 show the coagulation parameters before and after consuming the ATO, ATRA/chemotherapy. As mentioned in these studies, the parameters are in a high score, especially in D-dimer. The reduction of platelet (PLT) count demonstrates that they probably have been consumed in DIC or similar coagulopathy conditions. Jhang Y et al. study has demonstrated that the platelet count and fibrinogen levels in APL patients were low and inversely these patients had an evaluated rate in D-dimer, PT and APTT ^   21 ^. Other studies such as Hou J et al. and Xu F et al. acknowledged that the incidence of APL and its role in DIC happening caused early death in patients ^22^^,^^7^, so that the treatment should be launched immediately to save or at least lessen the danger of DIC. In these studies, the treatment was done with All-trans-retinoic Acid (ATRA), Arsenic Trioxide (ATO) and/or chemotherapy ^7^^,^^21^^–^^26^ . Jhang Y et al. study illustrated that after the treatment on 103 patients, on day 0 to day 29, the APTT was in the normal range and the Fibrinogen in 10 days and PT test in 4 days came to normal range. After that, they concluded that ATO solely could not accelerate the recovery, but when it comes to ATRA+chemotherapy can alleviate the burden of the blood coagulation. They claimed that the recovery of APL patients for the DIC scores was due to the blood products injection^   22 ^. In Hou J et al. study the treatment was based on single-agent ATO and unlike to the Jhang Y et al, 180 (83.3%) of patients had complete hematological recovery and the rest of 36 (16.7%) died because of the different reasons^   7 ^. Xu F et al. study included 212 APL patients, of whom 49 patients (Age range: 15-84 years) were removed from the study because of death. The rest of 163 patients, consisting of 91 males and 72 females, achieved a complete remission with ATRA/ATO and chemotherapy. Xu F et al. study compared different parameters in early death (ED) at low risk, intermediate risk and high risk for those 49 patients ^   7 ^. 

The study of Zho HH et al. contained 83 hospitalized patients with age range 15-59 divided into two “ATO” and “RIF” groups. 38 patients of the present study have participated in the ATO group. More than 90% of patients had elevated D-dimer and hypofibrinogenemia^2^^,^^23^ .

After then, 3 case study articles were checked in relation to our study. The first study included a 56-year old woman with APL. At first, she received ATRA + chemotherapy for 3 times and achieved complete remission, and then the therapy was continued with ATO^   25 ^. The second case report included a 78-year old Hispanic man with some diseases such as type 2 diabetes mellitus, history of Alzheimer and hypertension because of the CML. Initially, he was under the Imatinib treatment and then Dasatinib. 7 years later, the result of complete blood count (CBC) showed abnormally elevated promyelocytes, and then characterized that besides t(9;22) for CML he had t(15;17)(PML-RARA), and his treatment started with ATRA/ATO. The last study included a 60-year old Japanese man with APL that he received ATRA and chemotherapy. But after 4 months he did not achieve complete remission, and then therapy started with ATO^   24 ^.

**Table 2:** Characteristics of APL patients in included studies

**Study**	**NO.**	**Induction**	**Age (years)**	**Sex (M-F)**	**Translocation**
Zhang Y, *et al* (2016)	103	ATRA, ATO, Chemo	14-74	60-43	t(15;17)
Hou J, *et.al* (2017)	216	ATO	7-80	105-111	t(15;17)
H-H. Zhu, *et al* (2018)	38	ATO	15-59	23-15	t(15;17)
Xu F, *et al* (2017)	163	ATRA, ATO, Chemo	15-54	91-72	t(15;17)
H. Agis, *et al* (1999)	1	ATRA, ATO	56	0-1	t(15;17)
T.A. Colvin, *et al* (2018)	1	ATRA, ATO	78	1-0	t(15;17)
Ishitsuka, *et. al* (2004)	1	ATO	60	1-0	t(15;17)

M: Male, F: Female, ATRA: All Trans Retinoic Acid, ATO: Arsenic Trioxide, Chemo: Chemotherapy

**Table 3:** D-dimer laboratory parameter before and after treatment in APL patients

**Study**	**D-dimer (ng/ml) Before**	**D-dimer (ng/ml) After**	**P**
Zhang Y, et al (2016)	19900 (8000-33100)	1000 (600-1900)	˂0.001
Hou J, et.al (2017)	4400 (0-80000)	4800 (200–80000)	˂0.05
H-H. Zhu, et al (2018)	1648.26 (277-6503)	≈ 200 (50-700)	˂0.05
Xu F, et al (2017)	1741.5 (90-36370)	1289.5 (1.5–177,500)	0.201
H. Agis, et al (1999)	14600	≈ 750	-
T.A. Colvin, et al (2018)	10100	≈ 300	-
Ishitsuka, et. al (2004)	68490	137300	-

**Table 4:** Prothrombin Time laboratory parameter before and after treatment in APL patients

**Study**	**PT (sec)** **Before**	**PT (sec)** **After**	**P**
Zhang Y, et al (2016)	16.7 (14.8-19.8)	13.5 (12.4-14.6)	˂0.001
Hou J, et.al (2017)	-	-	-
H-H. Zhu, et al (2018)	14.16 (11-20.1)	≈ 12.5 (11-14)	˂0.05
Xu F, et al (2017)	14	13	˂0.01
H. Agis, et al (1999)	-	-	-
T.A. Colvin, et al (2018)	13.4	-	-
Ishitsuka, et. al (2004)	13.2	-	-

PT: Prothrombin Time

**Table 5:** Activated Partial Thromboplastin Time laboratory parameter before and after treatment in APL patients

**Study**	**APTT (sec)** **Before**	**APTT (sec)** **After**	**P**
Zhang Y, et al (2016)	34.4 (28.7-40.2)	34.7 (27.7-44.1)	0.883
Hou J, et.al (2017)	-	-	-
H-H. Zhu, et al (2018)	27.95 (17.4–37)	31 (19.1-39)	˂0.05
Xu F, et al (2017)	40	33	0.35
H. Agis, et al (1999)	-	-	-
T.A. Colvin, et al (2018)	30.5	-	-
Ishitsuka, et. al (2004)	-	-	-

APTT: Activated Partial Thromboplastin Time

**Table 6:** Platelet laboratory parameters before and after treatment in APL patients

**Study**	**Platelet ×10** ^3^ **/µl** **Before**	**Platelet ×10** ^3^ **/µl** **After**	**P**
Zhang Y, et al (2016)	25 (13-40)	45 (31-104)	˂0.001
Hou J, et.al (2017)	22 (1-331)	20 (40-73)	˂0.05
H-H. Zhu, et al (2018)	44.11 (10-164)	≈ 110 (40-190)	˂0.05
Xu F, et al (2017)	28 (5.00–159.00)	28 (6–159)	0.14
H. Agis, et al (1999)	95	≈ 100	-
T.A. Colvin, et al (2018)	6	41	-
Ishitsuka, et. al (2004)	58	≈ 9	-

**Table 7:** White Blood Cell laboratory parameter before and after treatment in APL patients.

**Study**	**WBC ×10** ^3^ **/µl** **Before**	**WBC ×10** ^3^ **/µl** **After**	**P**
Zhang Y, et al (2016)	-	-	-
Hou J, et.al (2017)	2.7 (0.3–211.1)	2.8 (0.4–42.1)	0.586
H-H. Zhu, et al (2018)	7.46 (0.31–45)	˂10	
Xu F, et al (2017)	2.81 (0.41–170.49)	36.55 (10.18–170.49)	˂0.1
H. Agis, et al (1999)	≈ 3.5	≈ 3	-
T.A. Colvin, et al (2018)	1.03	1.46	-
Ishitsuka, et. al (2004)	6.6	54	-

WBC: White Blood Cell

**Figure 2:**
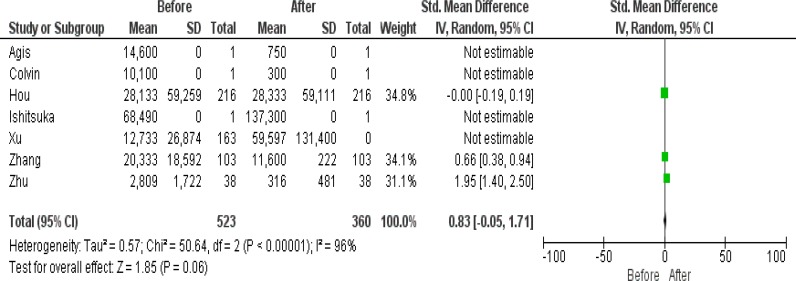
Forrest plot of D.dimer before and after treatment

**Figure 3:**
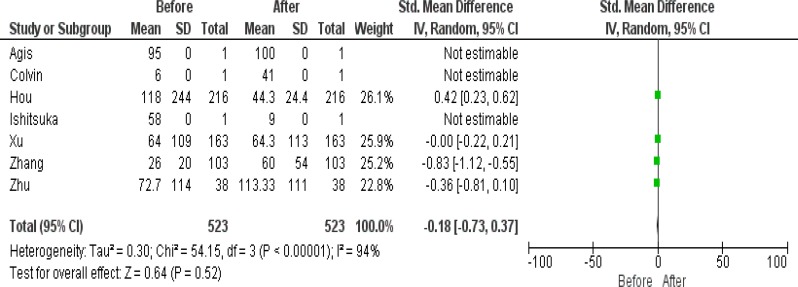
Forrest plot of Platelet before and after treatment

**Figure 4:**
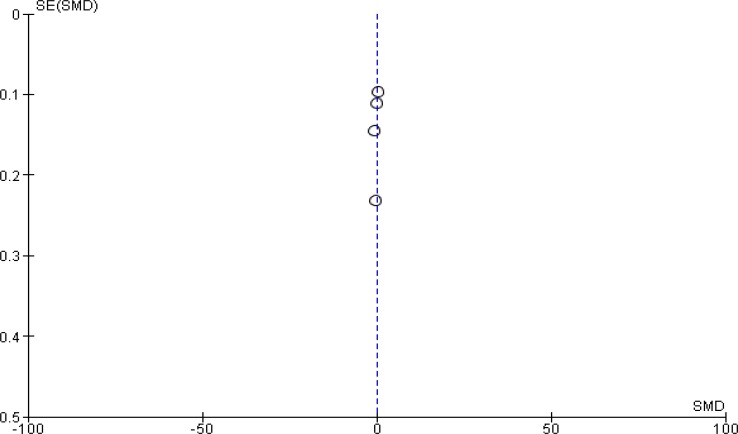
Funnel plot of involved studies (Zhang Y, et al (2016) Hou J, et.al (2017) H-H. Zhu, et al (2018) Xu F, et al (2017))

## Discussion

This study was on patients involved with acute promyelocytic leukemia. All patients had t(15;17)(PML;RARA). The incidence of coagulopathies is higher in APL patients. Arsenic trioxide (ATO) is a toxic therapeutic method for cells. ATO often is used after ATRA and/or chemotherapy. The analysis of D-dimer parameter illustrated that 58.50% (n=306) (p <0.05) of cases were reduced after treatment. The analysis PT test showed that all cases (p <0.05) were decreased, but the PTT test was increased in 46.55% (n=142) of patients. The study of platelet count in 58.69% (n=307) (p <0.05) were recovered after treatment. APL treatment with ATO+ATRA has recovery effect on WBC and PLT count (Tables 6, 7). Many studies showed that treatment with ATO without ATRA and chemotherapy seems not to be sufficient for APL patients and it has some bad side effects ^7^^,^^21^^–^^25^ . As results showed that the ATO does not have the potential to be used solely or at the frontline of the therapy procedure, it may be better to be prescribed with ATRA and chemotherapy or at least be used at the end-stage. The ATO treatment with or without ATRA or chemotherapy does not have a significant reduction effect on DIC parameters (D-dimer, PT and APTT) (Tables 3,4,5). ATO like other anti-cancer therapies has the potential to damage other cells because after using ATO, patients get in trouble with hypoplasia in the bone marrow and finally in peripheral blood, so because of these risky effects the physicians often prescribe ATO in a low dose.

## References

[1] De Kouchkovsky I, Abdul-Hay M (2016). Acute myeloid leukemia: a comprehensive review and 2016 update. Blood Cancer J.

[2] Li J, Chen P, Sinogeeva N (2002). Arsenic trioxide promotes histone H3 phosphoacetylation at the chromatin of CASPASE-10 in acute promyelocytic leukemia cells. J Biol Chem.

[3] Melo RA, de Vasconcellos JF, Melo FC (2006). PML-RARalpha fusion gene transcripts and biological features in acute promyelocytic leukemia patients. Clin Lab Haematol.

[4] Chang H, Kuo MC, Shih LY (2012). Clinical bleeding events and laboratory coagulation profiles in acute promyelocytic leukemia. Eur J Haematol.

[5] Coombs CC, Tavakkoli M, Tallman MS (2015). Acute promyelocytic leukemia: where did we start, where are we now, and the future. Blood Cancer J.

[6] Lee HJ, Park HJ, Kim HW (2013). Comparison of laboratory characteristics between acute promyelocytic leukemia and other subtypes of acute myeloid leukemia with disseminated intravascular coagulation. Blood Res.

[7] Xu F, Wang C, Yin C (2017). Analysis of early death in newly diagnosed acute promyelocytic leukemia patients. Medicine (Baltimore).

[8] Shahmarvand N, Oak JS, Cascio MJ (2017). A study of disseminated intravascular coagulation in acute leukemia reveals markedly elevated D-dimer levels are a sensitive indicator of acute promyelocytic leukemia. Int J Lab Hematol.

[9] Vignoli A, Marchetti M, Falanga A (2018). Acute promyelocytic leukemia cell adhesion to vascular endothelium is reduced by heparins. Ann Hematol.

[10] Jácomo RH, Santana-Lemos BA, Lima AS (2012). Methionine-induced hyperhomocysteinemia reverts fibrinolytic pathway activation in a murine model of acute promyelocytic leukemia. Blood.

[11] Song LX, Lu HY, Chang CK (2014). Cerebral venous and sinus thrombosis in a patient with acute promyelocytic leukemia during all-trans retinoic acid induction treatment. Blood Coagul Fibrinolysis.

[12] Lee HJ, Kim DH, Lee S (2015). Analysis of factors affecting hemorrhagic diathesis and overall survival in patients with acute promyelocytic leukemia. Korean J Intern Med.

[13] Lou Y, Suo S, Tong H (2016). Hypofibrinogenemia as a clue in the presumptive diagnosis of acute promyelocytic leukemia. Leuk Res.

[14] Cao M, Li T, He Z (2017). Promyelocytic extracellular chromatin exacerbates coagulation and fibrinolysis in acute promyelocytic leukemia. Blood.

[15] David S, Mathews V (2018). Mechanisms and management of coagulopathy in acute promyelocytic leukemia. Thromb Res.

[16] Daver N, Kantarjian H, Marcucci G (2015). Clinical characteristics and outcomes in patients with acute promyelocytic leukaemia and hyperleucocytosis. Br J Haematol.

[17] Wang P, Zhang Y, Yang H (2018). Characteristics of fibrinolytic disorders in acute promyelocytic leukemia. Hematology.

[18] Shen ZX, Shi ZZ, Fang J (2004). All-trans retinoic acid/As2O3 combination yields a high quality remission and survival in newly diagnosed acute promyelocytic leukemia. Proc Natl Acad Sci U S A.

[19] Mathews V, George B, Lakshmi KM (2006). Single-agent arsenic trioxide in the treatment of newly diagnosed acute promyelocytic leukemia: durable remissions with minimal toxicity. Blood.

[20] Emadi A, Gore SD (2010). Arsenic trioxide - An old drug rediscovered. Blood Rev.

[21] Zhang Y, Wu S, Luo D (2016). Addition of Arsenic Trioxide into Induction Regimens Could Not Accelerate Recovery of Abnormality of Coagulation and Fibrinolysis in Patients with Acute Promyelocytic Leukemia. PloS One.

[22] Hou J, Wang S, Zhang Y (2017). Causes and prognostic factors for early death in patients with acute promyelocytic leukemia treated with single-agent arsenic trioxide. Ann Hematol.

[23] Zhu HH, Guo ZP, Jia JS (2018). The impact of oral arsenic and all-trans-retinoic acid on coagulopathy in acute promyelocytic leukemia. Leuk Res.

[24] Ishitsuka K, Shirahashi A, Iwao Y (2004). Bone marrow necrosis in a patient with acute promyelocytic leukemia during re-induction therapy with arsenic trioxide. Eur J Haematol.

[25] Agis H, Weltermann A, Mitterbauer G (1999). Successful treatment with arsenic trioxide of a patient with ATRA-resistant relapse of acute promyelocytic leukemia. Ann Hematol.

[26] Soignet SL (2001). Clinical Experience of Arsenic Trioxide in Relapsed Acute Promyelocytic Leukemia. Oncologist.

[27] Mathews V, George B, Lakshmi KM (2006). Single-agent arsenic trioxide in the treatment of newly diagnosed acute promyelocytic leukemia: durable remissions with minimal toxicity. Blood.

